# Amino acid substitutions in specific proteins correlate with farnesol unresponsiveness in *Candida albicans*

**DOI:** 10.1186/s12864-023-09174-y

**Published:** 2023-03-01

**Authors:** Sima Mohammadi, Annie Leduc, Steve J. Charette, Jean Barbeau, Antony T. Vincent

**Affiliations:** 1 Département des sciences animales, Faculté des sciences de l’agriculture et de l’alimentation, Université Laval, Pavillon Paul-Comtois, 2425 rue de l’Agriculture, G1V 0A6 Quebec City, QC Canada; 2 Institut de biologie intégrative et des systèmes, Université Laval, Quebec City, QC Canada; 3 Département de stomatologie, Faculté de Médecine Dentaire, Université de Montréal, Montreal City, QC Canada; 4 Centre de recherche de l’Institut universitaire de cardiologie et de pneumologie de Québec, Quebec City, QC Canada; 5 Département de biochimie, de microbiologie et de bio-informatique, Université Laval, Quebec City, QC Canada

**Keywords:** *Candida albicans*, Farnesol, Amino acid substitutions, Genomics, Evolution

## Abstract

**Background:**

The quorum-sensing molecule farnesol, in opportunistic yeast *Candida albicans*, modulates its dimorphic switch between yeast and hyphal forms, and biofilm formation. Although there is an increasing interest in farnesol as a potential antifungal drug, the molecular mechanism by which *C. albicans* responds to this molecule is still not fully understood.

**Results:**

A comparative genomic analysis between *C. albicans* strains that are naturally unresponsive to 30 µM of farnesol on TYE plates at 37 °C versus responsive strains uncovered new molecular determinants involved in the response to farnesol. While no signature gene was identified, amino acid changes in specific proteins were shown to correlate with the unresponsiveness to farnesol, particularly with substitutions in proteins known to be involved in the farnesol response. Although amino acid changes occur primarily in disordered regions of proteins, some amino acid changes were also found in known domains. Finally, the genomic investigation of intermediate-response strains showed that the non-response to farnesol occurs gradually following the successive accumulation of amino acid changes at specific positions.

**Conclusion:**

It is known that large genomic changes, such as recombinations and gene flow (losses and gains), can cause major phenotypic changes in pathogens. However, it is still not well known or documented how more subtle changes, such as amino acid substitutions, play a role in the adaptation of pathogens. The present study shows that amino acid changes can modulate *C. albicans* yeast’s response to farnesol. This study also improves our understanding of the network of proteins involved in the response to farnesol, and of the involvement of amino acid substitutions in cellular behavior.

**Supplementary Information:**

The online version contains supplementary material available at 10.1186/s12864-023-09174-y.

## Background

The yeast *Candida albicans* is normally a harmless commensal organism [1]. However, it can also be an opportunistic fungal pathogen in humans, causing a wide range of diseases from superficial mucosal infections to life-threatening systemic disorders [2, 3]. For example, *C. albicans* is the main causative agent of thrush in up to 95% of cases of oral yeast infections [4]. In addition, vulvovaginal candidiasis is a common mucosal infection caused mainly by *C. albicans*, which afflicts roughly 75% of women at least once in their lifetime [5]. Depending on the environmental conditions, *C. albicans* can grow in three main cellular morphologies: yeast as ovoid budding cells, hyphae as branching filamentous cells, and pseudohyphae as constricted chains of yeast cells. The ability of *C. albicans* to colonize or cause an infection depends on several virulence factors and traits, such as biofilm formation and the dimorphic switch between yeast and hyphal forms [6]. For several pathogens, including *C. albicans*, the formation of biofilms allows the yeast to get past host defenses, which can cause recalcitrant infections [7–10].

Quorum sensing (QS) is a strategy used by many microorganisms to assess population density through the production and sense of autoinducers [11]. QS in fungi is particularly important since it controls several intra- and inter-species mechanisms and behaviors, such as morphogenesis, sexual differentiation, and virulence [12, 13]. *C. albicans* is known to produce various QS molecules that regulate the morphogenetic process [11, 14]. Fungi and yeasts can also alter host immune cell recognition and response with QS molecules [15], but QS molecules with above-threshold concentrations can induce fungal apoptosis [13].

Farnesol, a precursor of the isoprenoid sterol that regulates germination, is the best-described QS molecule produced by *C. albicans* [16]. Farnesol has been shown to inhibit the yeast-to-mycelium conversion when it accumulates beyond a threshold level, leading to the yeast actively budding without influencing cellular growth rates [17]. However, farnesol is unable to halt the elongation of pre-existing hyphae [18, 19]. The inhibitory effects of farnesol on hyphal transition and biofilm formation [20] demonstrated that the antibiofilm properties of farnesol are potential preventive strategies [21].

Studying the effect of farnesol on biofilm formation is complex, because farnesol inhibits hyphal formation regulating several genes in the cyclic AMP signaling pathway [22]. Although the mechanisms of response to farnesol have never been characterized thoroughly, studies have shown that various genes are involved in the resistance of *C. albicans* biofilms to antifungals in the presence or absence of farnesol [23, 24].

In this study, by screening a library of clinical *C. albicans* strains that either do not, or only partially respond to farnesol, and thus continue to produce hyphae in presence of the molecule, we discovered that some amino acid substitutions in specific proteins correlate with the alteration of the normal response to farnesol. The present study showed that some molecular determinants may participate in the response to farnesol in *C. albicans*, and demonstrated that subtle mutations, such as amino acid changes, can have major phenotypic repercussions in pathogens.

## Results

**Fig. 1:**
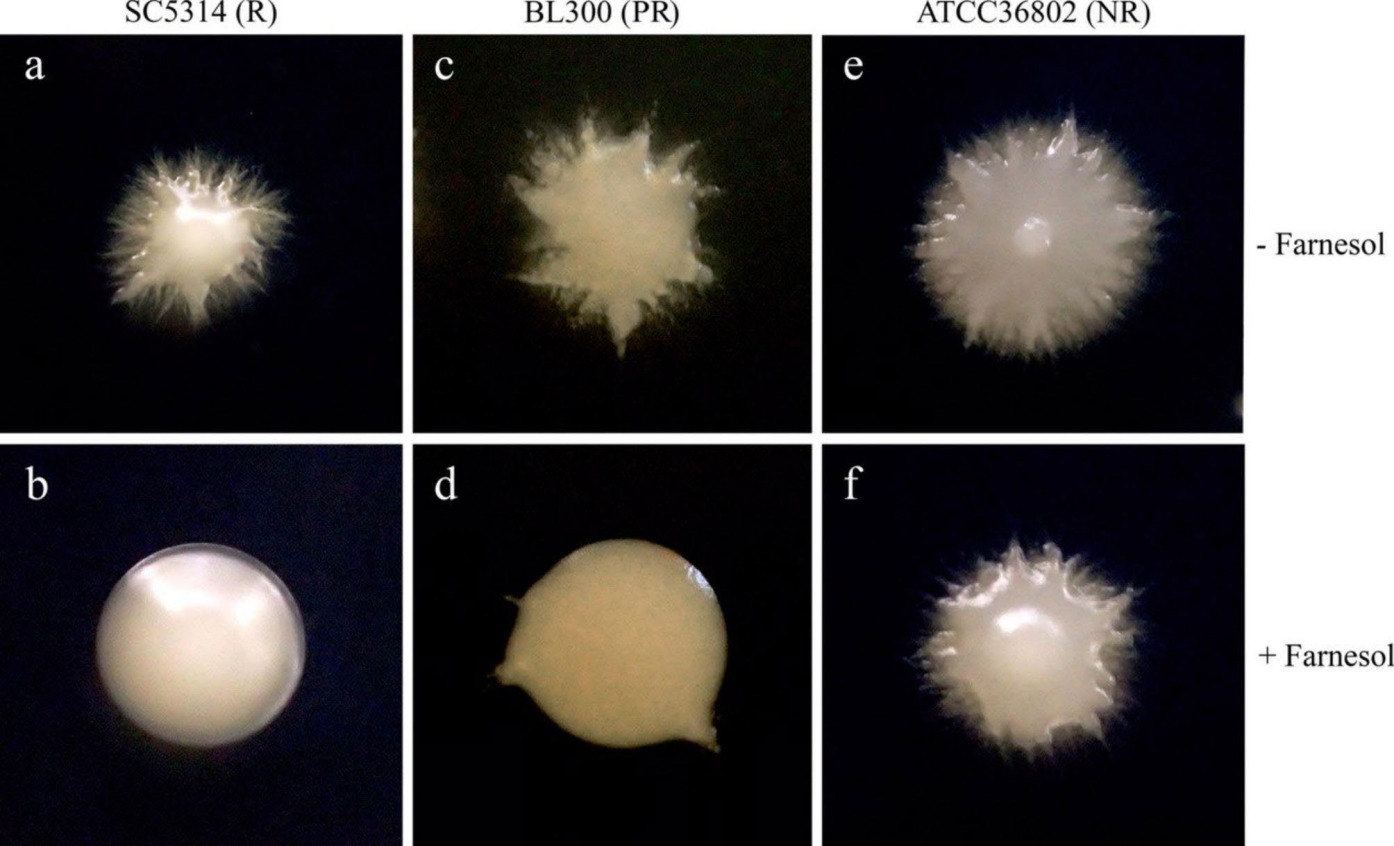
Three different phenotypes of *C. albicans* strains with or without 30 µM concentrations of farnesol. In SC5314, hyphal production was inhibited when 30 µM of farnesol is added to the solid medium (a,b). BL300 demonstrated partial hyphal production in presence of 30 µM farnesol (c,d). ATCC36802 did not respond to farnesol (e,f)

Since yeast-to-hypha growth is a key factor in *C. albicans* virulence, we tested the effect of farnesol on filamentous growth in different strains. Under the growth conditions used here, we found three different phenotypes of *C. albicans* strains in the presence of 30 µM of farnesol, a concentration close to the one produced naturally by *C. albicans* [25], after 48 h (Fig. 1). In SC5314, BL007, BL077, and BL273 strains hypha formation was inhibited at 30 µM of farnesol; the colonies adopt a smooth phenotype, which is the expected phenotype for strains that are responsive (R) to the farnesol molecule [26]. Three other strains (ATCC36802, HM1, and BL266) did not respond to farnesol and kept the hairy phenotype (non-responsive, NR). Interestingly, six strains (JMN070, BL167, BL288, BL300, BL296, and BL152) adopt a partial phenotype (partially responsive, PR) that showed some extent of hyphae growth.

To better understand the molecular determinants involved in the response to farnesol in *C. albicans*, the DNA of the studied strains was sequenced. Assembly and annotation revealed GC content, genome size, and the number of proteins similar to what was previously described for *C. albicans* and other species of the *Candida* genus (Table 1) [27–29].

**Table 1:** General information on genome assembly and annotation of *C. albicans* strains

Strains	Contigs	Total length	GC (%)	N50	Number of proteins	BioSample
Responsive (R)						
SC5314	8	14,282,666	33.46	2,231,883	6030	SAMN02953594
BL007	257	14,518,123	33.43	209,255	5869	SAMN31242278
BL077	279	14,665,524	33.45	161,380	5904	SAMN31242279
BL273	291	14,456,289	33.42	111,779	5840	SAMN31242283
Non-responsive (NR)						
ATCC36802	262	14,551,640	33.43	163,339	5877	SAMN31242277
HM1	234	14,456,289	33.44	201,779	5736	SAMN31242287
BL266	339	14,665,214	33.44	98,646	5982	SAMN31242282
Partially responsive (PR)						
JMN070	310	14,433,412	33.40	138,611	5838	SAMN31242288
BL167	350	14,441,282	33.39	95,450	5863	SAMN31242281
BL288	264	14,385,958	33.42	178,681	5853	SAMN31242284
BL300	333	14,576,599	33.39	163,922	5903	SAMN31242286
BL296	252	14,494,849	33.42	219,301	5861	SAMN31242285
BL152	342	14,707,636	33.43	162,594	5942	SAMN31242280

**Fig. 2:**
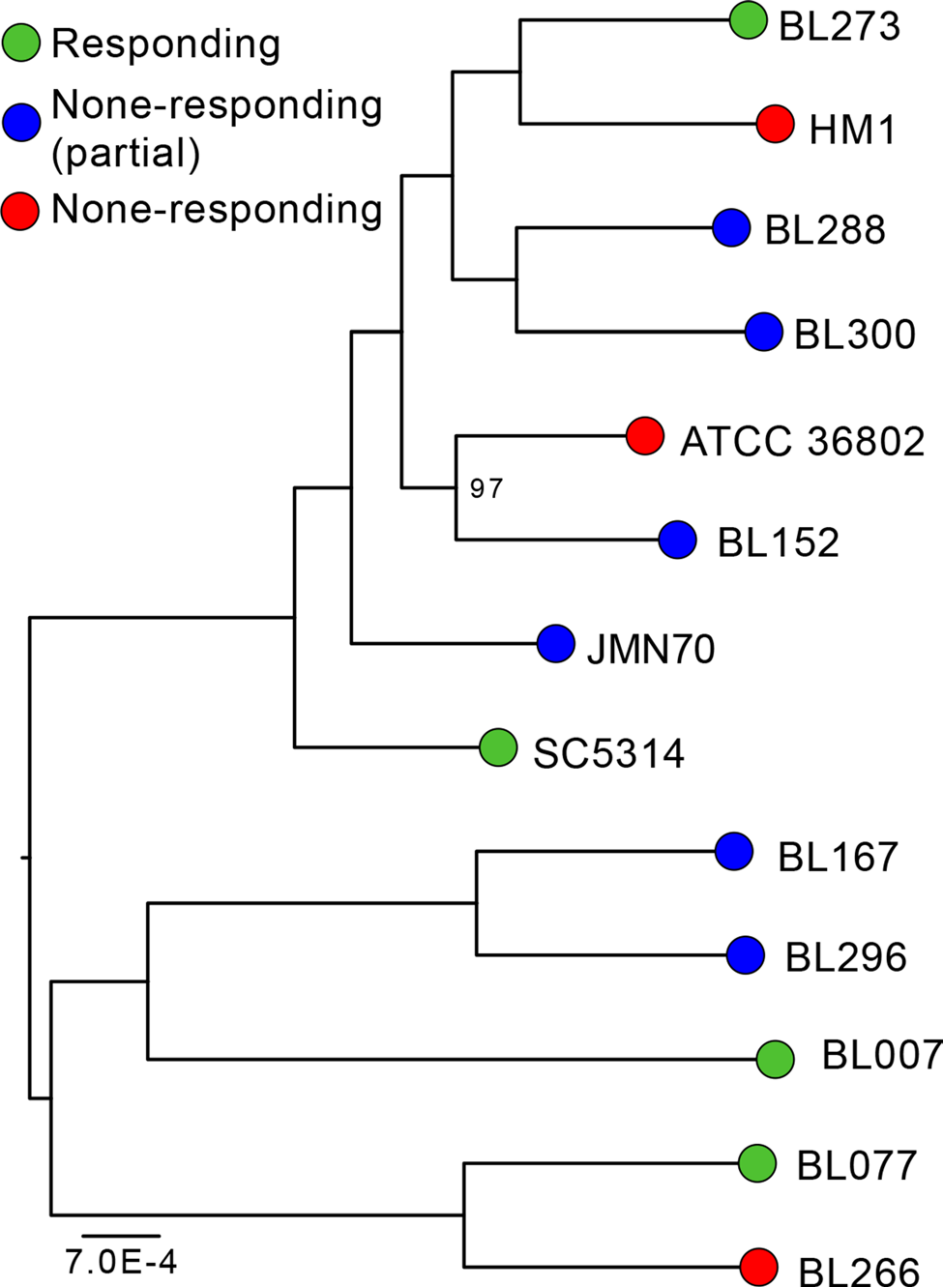
A maximum likelihood tree of the *C. albicans* strains studied. Different strains with distinct levels of phenotypic response to farnesol are represented as colorful circles: green, complete phenotype response to farnesol (R); blue, partial phenotype response to farnesol (PR); red, no phenotype response to farnesol (NR). The boostraps values are all 100, except for one node which has a support of 97 (shown in the figure)

Sequences were used to examine the phylogenetic relationships of *C. albicans* strains (Fig. 2). The phenotypes (level of response to farnesol) are not monophyletic; the strains are scattered all over the tree. This analysis thus suggests that the difference in the level of response to farnesol is the result of convergent evolution, and was not transmitted vertically among the strains studied. Also, the lengths of the branches are relatively uniform, showing that there is no obvious correlation between the rate of evolution and the level of response to farnesol.

**Fig. 3:**
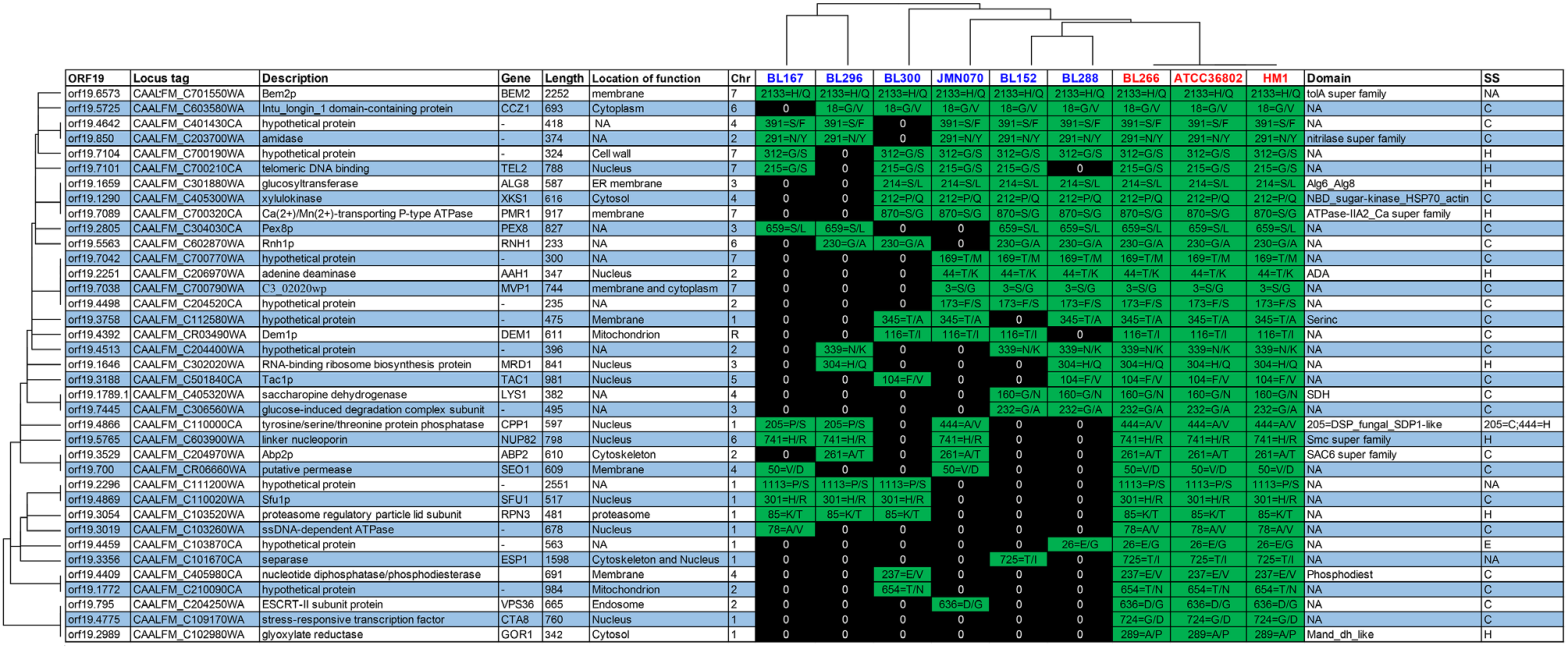
A list of 38 amino acid substitutions was identified in 37 proteins correlating with unresponsiveness to farnesol in NR (red) and PR (blue) strains of *C. albicans*. The information is given according to the reference strain SC5314. Every column in the center of the matrix indicates one strain of *C. albicans* and the green and black boxes show the presence and absence of amino acid substitutions respectively. NR and PR strains are categorized based on the amounts of amino acid substitution that are detected among them. The white and blue rows show information about detected proteins such as name, description, length, location of the activity, chromosomal location, conserved domains, and secondary structures (SS); (H = helix, C = coil, S = strand)

A sequence homology search was performed to determine whether the unresponsiveness to farnesol could be caused by the presence or absence of one or more genes. By comparing sequences from the responsive and non-responsive strains, it was not possible to find any gene that could be a signature of the phenotype. Similarly, no point mutations in regulatory regions were found only in the genomes of non-responsive strains. A recent bioinformatics tool, CAPRIB [30], was used to verify if amino acid changes correlated with unresponsiveness to farnesol. While no marker gene was identified, a total of 38 positions in proteins of strains that respond to farnesol were found to be systematically different in strains that do not respond (Fig. 3). The 38 positions are found on 37 proteins (one of the proteins has two mutations). By investigating the sequences of strains that partially respond to farnesol, several of the 38 amino acid changes were found. However, compared to strains that have complete unresponsiveness to farnesol (NR), no partially responsive strains (PR) have all 38 amino acid changes.

Several mutations provide strong evidence for the involvement of detected proteins in the level of response to farnesol. For example, in an earlier study, ALG8 was described as a gene whose activation resulted in sensitivity to farnesol [31]. In the three strains that do not respond to farnesol and in four strains with a partial phenotype, the same substitution in Alg8p was observed. This reinforces the idea that ALG8 plays an important role in the pathway of response to farnesol.

As expected, some proteins have clear roles in hypha morphology and hypha elongation rate as farnesol has direct effects on the hypha growth of *C. albicans*. For example, the PMR1 gene induced a decrease in the rate of hypha elongation in yeast [32]. Therefore, identifying an amino acid substitution in Pmr1p suggested a new role for this protein as an effector of the morphological response to farnesol. In another example, a previous study showed that CCZ1 was required for filamentous development and virulence in *C. albicans* and was a promising target for antifungal drug development [33]. The presence of Ccz1p as a candidate protein for the response to farnesol indicated other aspects of its function. An amino acid change was also detected in Sfu1p, a protein involved in RNA biosynthesis, and whose gene is known to be downregulated in the presence of farnesol in *Candida auris* [34], a species close to *C. albicans*.

Only one amino acid change, in the Bem2p protein, is found in all NR or PR strains. Earlier studies showed that Bem2p plays significant roles in morphogenesis checkpoints, and the cooperation between Bem2p and Bem3p is essential for bud emergence [35, 36]. In contrast, two mutations in two proteins (Cta8p and Gor1p) are exclusively present in NR strains and not at all in PR strains. Cta8p and Gor1p are involved in stress responses [37] and there is currently no obvious evidence for the relevant roles of these proteins in the pathway of response to farnesol.

Evidence shows that Cpp1p of *C. albicans* represses hyphal gene expression and regulates morphological changes by blocking the yeast-to-hyphal transition [38]. Regardless of the clear role of Cpp1p in response to farnesol, among candidate proteins, only Cpp1p showed two different amino acid changes in PR strains.

The relation between the detected amino changes and the conserved domains of proteins can provide insight into the structural involvement of the mutations. Among the 38 substitutions that were identified, 13 were in conserved domains which indicate functionally important regions (Fig. 3). Protein function is directly related to the structure of that protein, and among the 38 detected mutations, 24 (63%) are observed at coil secondary structures. In addition, the proteins with amino acid changes are in every chromosome of *C. albicans*, although chromosome 7 has the most changes (see Additional file 1), with respect to the total number of proteins by chromosome.

**Fig. 4:**
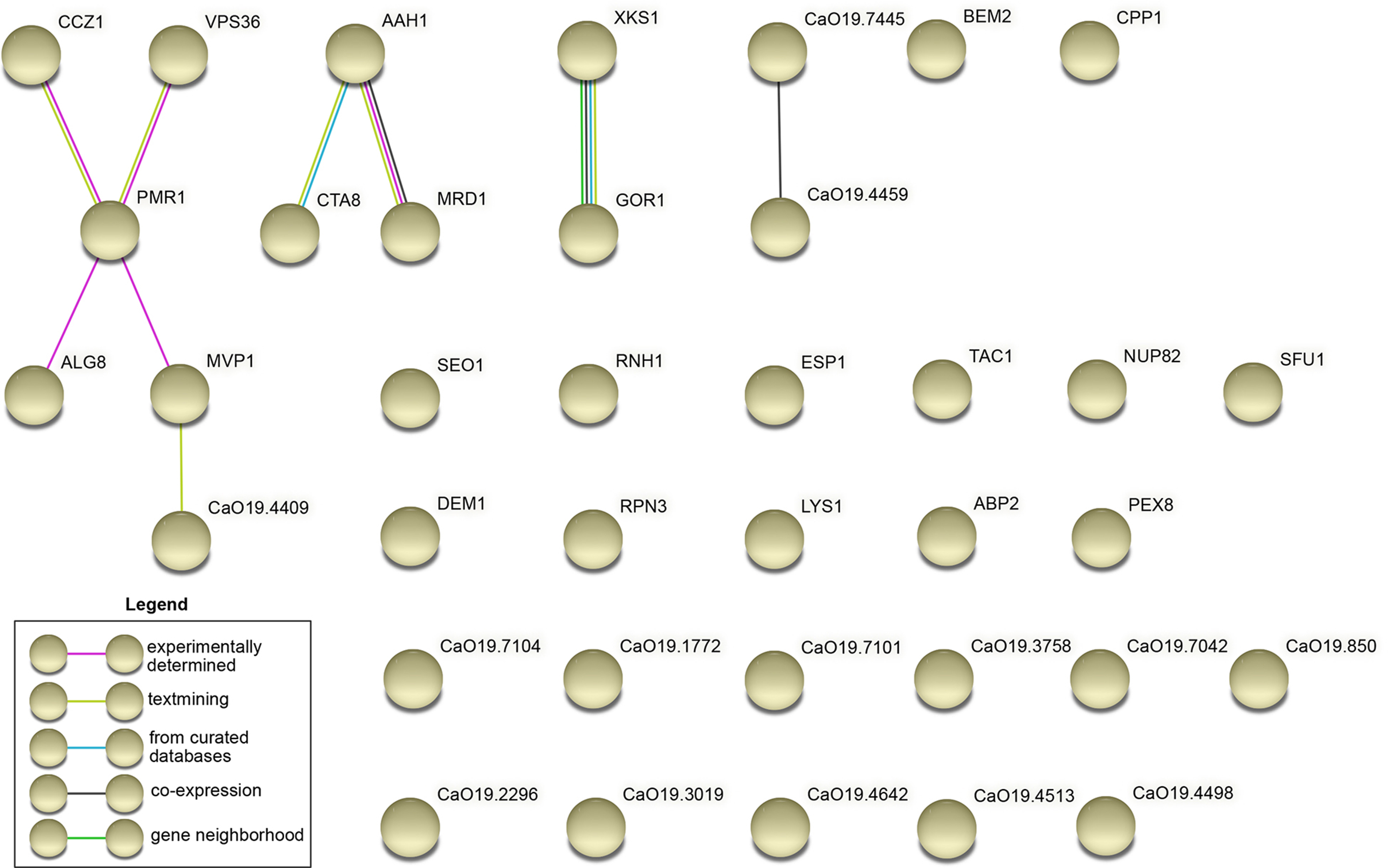
A STRING analysis showing putative relationships between genes found mutated in NR strains

The connections of genes that contain the mutations were analyzed through a STRING analysis. As shown in Fig. 4, some gene interactions were revealed, such as associations of ALG8, already known to be involved in response to farnesol, with PMR1, CCZ1, and MVP1. This network may indicate a part of the pathway of response to farnesol.

## Discussion

It has been demonstrated that farnesol acts as a quorum-sensing molecule that inhibits filamentation and biofilm formation in *C. albicans* [17]. Although studies have revealed certain molecular determinants involved in the production and response to farnesol [23, 26], the entire protein interaction network and the way *C. albicans* modulates its genome to respond to this molecule remain largely unknown.

We investigated genome sequences of clinical strains that respond and do not respond to farnesol to better understand, by comparative genomics, the mechanism of response to this molecule. Clinical isolates are of great interest since they reflect what happens in real life, not genetic manipulation in the laboratory. It is known that genomic plasticity, and gene flow in general, play a determining role in the diversification and evolution of *C. albicans* [39]. Surprisingly, according to our results, no marker gene for response or non-response to farnesol was found. This suggests that the loss of genes involved in the response to farnesol is not the mechanism preferentially selected by evolution to modulate the response for this molecule. However, as reviewed elsewhere [40], *C. albicans*, in addition to being able to modulate its gene repertoire, has a myriad of other mechanisms that allow it to modify its genome and thus adapt to various environments and adjust its virulence.

*C. albicans* has a compact genome for a eukaryote (~ 36% of coding) with strong purifying selection to preserve the integrity of the coding regions of point mutations [39]. Despite this, it has been found that non-synonymous mutations occur uniformly across the genome [41] and that genes that encode for cell surface proteins are conducive to a higher rate of mutation [42, 43]. While no gene is a marker of response or non-response to farnesol, it was possible to identify 38 amino acid substitutions that correlate with the level of response. Moreover, among the 37 proteins in which amino acid substitutions were detected, some are known to be involved in farnesol response. This is the case for ALG8, which is known to be involved in the farnesol sensitivity gene network interaction [31]. Interestingly, ALG8 is connected in a network with other genes, such as PMR1, CCZ1, MVP1, VPS36, and CAALFM_C405980CA, for which proteins have amino acid substitutions (Fig. 4). The genes PMR1 and CCZ1 have obvious roles in limiting hyphae development [33, 44] and may thus also be involved in farnesol response.

In *C. albicans*, farnesol affects the transcription level of several genes [45, 46]. Interestingly, conserved amino acid substitutions were found in three transcription proteins (Sfu1p, C3_02020wp (encoded by MRD1), Nup82p). The gene SFU1 is already known to downregulate in the presence of farnesol in *C. auris* [45]. Besides, *C. albicans* globally up-regulates its cellular metabolism in response to farnesol [47, 48]. Proteins found by the present study, such as those encoded by GOR1, ALG8, XKS1, AAH1, and LYS1, are also involved in cellular metabolism [49–53], suggesting that these proteins may impact farnesol responsiveness by having global effects on the cells.

Only one protein, Bem2p, have a conserved amino acid substitution in all strains that do not fully respond to farnesol. Two hypotheses could be proposed to explain the roles of the substitution in Bem2p for response to farnesol. On the first hand, the function of Bem2p is essential in controlling *C. albicans* virulence-pathogenesis in the farnesol responsiveness pathway. On the other hand, it is possible to postulate that this mutation must appear early in order to allow the other mutations. Contrarily, Cta8p and Gor1p are two proteins that showed amino acid substitutions just in NR strains, both of which may be essential for *C. albicans* to have complete farnesol resistance.

We expanded our mutational analysis of detected proteins by investigating conserved domains. There is evidence that mutations in conserved regions may be detrimental to the proteins [54, 55]. Some detected amino acid substitutions are located in conserved domains, such as for proteins Alg8p, Bem2p, Pmr1p, and Cpp1p. This suggests structural distortion of these proteins. In PR strains the presence of two distinct mutations in Cpp1p (Figs. 3 and 205P/S and 444 A/V) can be an example that several positions of a protein might be decisive for farnesol responsiveness. Also, 63% of the substituted residues found in this study were located in coil regions, a structure more resilient to mutations [56].

## Conclusion

These new findings about genotype-phenotype associations add to the understanding of *C. albicans* adaptation, virulence, and pathogenicity as a whole. Comparing the genomes of clinical strains that do not respond to farnesol did not reveal any gene that was a marker of response or non-response to farnesol. However, there are amino acid substitutions that correlate with the level of phenotypic farnesol response. This demonstrates that subtle mutations, such as amino acid substitutions, can cause important phenotypes in pathogens, as recently suggested in the bacteria of the genus *Mycobacterium* [30]. Among proteins with amino acid substitutions, Alg8p is a known gene that plays an obvious role in the *C. albicans* farnesol response. Also, the connection of ALG8 with some other genes, such as PMR, CCZ1, VPS36, and MVP1 introduces potential new roles for them in mediating the *C. albicans* response to farnesol. Therefore, this study provides new and valuable insights not only in our understanding of the molecular pathway of response to farnesol but also may lead to investigations of novel strategies for the development of antifungal drugs. Finally, as more genomes of *C. albicans* strains that naturally do not respond to farnesol are sequenced, the extent of the mechanisms allowing the response to this molecule will be known ever-more precisely.

## Methods

### Strains, culture and farnesol responsiveness assessment

The *C. albicans* strains used in this study are listed in Table 2. All strains were isolated from patients of Faculté de Médecine Dentaire clinics, Université de Montréal, and graciously donated by third parties to this project. Conservation of clinical strains were made by cryopreservation in glycerol at -80 °C. For the farnesol assay, strains were thawed on Sabouraud Dextrose Agar (SDA). For each strain, single colonies of *C. albicans* were cultivated for 24 h without agitation at 37 °C in TYE liquid media (For 1 L: 17 g trypticase-peptone, 3 g yeast extract, 5 g sodium chloride, 2.5 g sodium phosphate dibasic anhydrous) and 1.5% (W/V) of glucose, sterilized by filtration. For farnesol response assays, cells were pelleted and washed twice with sterile saline (NaCl 0.85% (W/V)). Approximately 250 cells, determined by hemacytometer or viable count on SDA, were plated on a TYE agar with 30 µM trans, trans-farnesol (Sigma, St-Louis, MO) diluted in methanol and incubated for 48 h at 37 ˚C with 2.5% CO_2_. A control condition with the same volume of methanol without farnesol was also used. Farnesol response assays were repeated three times in three different cultures and the morphology of every colony was surveyed. The strains were classified based on their phenotypic response in the presence of 30 µM farnesol. Strains with more than 90% of the smooth colony were considered responsive phenotypes, strains with a stable population of more than 90% hairy colonies (i.e., with hyphal production) were considered non-responsive, and strains with 10–90% of hairy colonies on the plate were considered partially responsive (Table 2).

**Table 2:** *Candida albicans* strains used in this study

Strains	Response	Gender	Age	Date of sampling	Information
				(M. D. Y.)	
SC5314	R			1984	clinical specimen
BL007	R	F	63	11.20.2001	pseudomembranous candidiasis. Taking Cromolyn and Lectopam
BL077	R	M	N/A	06.15.2009	pronounced gingival erythema
BL273	R	M	73	09.16.2016	lichen planus. Topical corticosteroid
ATCC 36,802	NR	N/A	N/A	N/A	ATCC catalog
HM1	NR	N/A	N/A	N/A	name for: heavy myceliated from Geneva, Swiss, Gift of Dre Noëlla Deslauriers, Université Laval
BL266	NR	F	21	06.17.2019	angular cheilitis. Pain at the corner of the lips
JMN070	PR	N/A	11	01.03.2018	Down Syndrome possible
BL167	PR	F	50	08.17.2015	erythema on left lateral surface of the tongue. Taking corticosteroid for lichen planus for 3 months
BL288	PR	F	77	01.08.2020	erythematous patches on the cheeks. Topical corticosteroids for lichen planus
BL300	PR	F	41	10.12.2021	white papules on a slightly erythematous base on the left cheek mucosa. Presence of depapillated area on the tongue
BL296	PR	F	88	04.28.2021	severe xerostomia and lichen planus
BL152	PR	F	67	11.25.2014	hyposialia and atrophy of the filiform papillae of the dorsal tongue

N/A: not available in medical file or literature; Gender: male (M) or female (F); Responsive (R): strain responsive to 30 µM farnesol, more than 90% of smooth colony was observed; Non-responsive (NR): strain presenting, in a stable way, more than 90% of hairy colonies on 30 µM farnesol; Partially responsive (PR): strain with variable response to farnesol between 10 and 90% of hairy colonies.

### Sequencing and bioinformatics analyses

*C. albicans* strains were grown on Sabouraud’s 2% dextrose agar at 30 ˚C for 72 h. Genomic DNA extraction of the 12 strains was performed using the DNeasy PowerSoil Pro extraction kit from QIAGEN, following the manufacturer’s instructions. The purified DNA samples were sequenced on an Illumina NovaSeq 6000 device by the Genome Quebec Centre of Expertise and Services (Montréal, Canada). The resulting sequencing reads of each strain were *de novo* assembled using MaSuRCA 4.0.9 [57]. The annotation was carried out using the funannotate pipeline 1.8.9 [58].

The sequences of the 13 strains of *C. albicans* (including the reference strain SC5314) were used to construct a molecular phylogeny. The complete pipeline used is described in detail elsewhere [59]. The differences are that in the present study GET_HOMOLOGUES 20,220,516 was used to find homology links and IQ-TREE 1.4.4 for phylogenetic reconstruction with 10,000 ultrafast boostraps. The final tree has been midpoint-rooted using FigTree version 1.4.3 (http://tree.bio.ed.ac.uk/software/figtree/). GET_HOMOLOGUES was also used to search for signature genes that could explain the non-response to farnesol.

The breseq tool version 0.37.1 [60] was used to identify point mutations in the regulatory regions of genes. The − 150 bp and + 150 bp regions of the genes were considered. The genome of strain SC5314 was used as a reference. The CAPRIB tool [30] was used to determine the amino acid changes that correlate with unresponsiveness to farnesol. However, BLASTP 2.12.0 was used with translated sequences annotated with funannotate instead of TBLASTN. Only proteins that share more than 85% identity and less than 1e^− 10^ were considered homologous. The SC5314 strain was used as a reference. Amino acid changes and strains were clustered according to a binary model in R.

The NCBI conserved domains database[61] was used to delineate the conserved domains of the studied proteins. PSIPRED 4[62] was used to generate secondary structure predictions and MEMSAT-SVM[63] to predict transmembrane regions of proteins. Functional links between proteins were detected by using STRING version 11.5 [64].

## Electronic supplementary material

Below is the link to the electronic supplementary material.

**Additional file 1. Figure S1.** The percentage of proteins with amino acid changes in every chromosome of the reference strain SC5314, with respect to the total number of proteins by chromosome.

## Data Availability

The genomic sequences were deposited at DDBJ/ENA/GenBank under the BioProject PRJNA889397.

## Abbreviations

QS: Quorum sensing
*C. albicans*: *Candida albicans*
DNA: Deoxyribonucleic acid
SDA: Sabouraud dextrose agar
TYE: Tryptone yeast extract
