# Supplementary material for: Amino acid substitutions in specific proteins correlate with farnesol unresponsiveness in Candida albicans
Source: BMC Genomics. 2023 Mar 1;24:93. doi: 10.1186/s12864-023-09174-y (PMC9979538; doi:10.1186/s12864-023-09174-y)
Supplement: Supplementary file 1 — Additional file 1. Figure S1. The percentage of proteins with amino acid changes in every chromosome of the reference strain SC5314, with respect to the total number of proteins by chromosome. [file 12864_2023_9174_MOESM1_ESM.docx]

**Amino acid substitutions in specific proteins correlate with farnesol unresponsiveness in *Candida albicans***

Sima Mohammadi^1,2^, Annie Leduc^3^, Steve J. Charette^2,4,5^, Jean Barbeau^3^, Antony T. Vincent^1,2,*^

1. Département des sciences animales, Faculté des sciences de l'agriculture et de l'alimentation, Université Laval, Quebec City, QC, Canada
2. Institut de biologie intégrative et des systèmes, Université Laval, Quebec City, QC, Canada
3. Département de stomatologie, Faculté de Médecine Dentaire, Université de Montréal, Montreal City, QC, Canada
4. Centre de recherche de l’Institut universitaire de cardiologie et de pneumologie de Québec, Quebec City, QC, Canada
5. Département de biochimie, de microbiologie et de bio-informatique, Université Laval, Quebec City, QC, Canada

*Correspondance: Antony T. Vincent, Ph. D.
Département des sciences animales
Pavillon Paul-Comtois 
2425 rue de l'Agriculture, Québec, QC G1V 0A6
Université Laval
antony.vincent@fsaa.ulaval.ca
1-418-656-2131 # 417949

**Figure S1.** The percentage of proteins with amino acid changes in every chromosome of the reference strain SC5314, with respect to the total number of proteins by chromosome. No mutations were detected in chromosome 5.
